# Cervicovaginal Microbiome Signatures Across Cervical Disease States: A Prospective Cross-Sectional Analysis

**DOI:** 10.3390/diagnostics16050753

**Published:** 2026-03-03

**Authors:** Alexandru Hamod, Oancea Mihaela, Mihaela Grigore, Ingrid-Andrada Vasilache, Ramona-Gabriela Ursu, Razvan Popovici, Ana-Maria Grigore, Ludmila Lozneanu, Dan-Constantin Andronic, Mitica Ciorpac, Manuela Ciocoiu

**Affiliations:** 1 Grigore T. Popa University of Medicine and Pharmacy, 700115 Iasi, Romaniamitica.ciorpac@umfiasi.ro (M.C.);; 2 IInd Department of Obstetrics Gynecology, Iuliu Haţieganu University of Medicine and Pharmacy Cluj-Napoca, 13 Emil Isac, 400023 Cluj-Napoca, Romania

**Keywords:** cervicovaginal microbiome, cervical dysplasia, *Lactobacillus*-to-anaerobe log-ratio, cervical cancer

## Abstract

**Background/Objectives**: The cervicovaginal microbiome has emerged as a critical determinant of cervical health. In this study, we aimed to characterize the cervicovaginal microbiome across a spectrum of cervical health states and to identify community-level features that distinguish invasive disease from precursor states. **Methods**: We analyzed cervicovaginal samples of 86 patients with normal epithelium, low-grade (LSIL) and high-grade (HSIL) intraepithelial lesions, and cervical carcinoma (CCU) and available HPV genotyping. Vaginal samples were subjected to full-length 16S rRNA gene sequencing and genus-level taxonomic profiles were generated using ONT-supported workflows. Microbiome diversity and composition were assessed using Aitchison-based beta-diversity, non-parametric testing, and PERMANOVA. Differential abundance was evaluated using ANCOM-BC2 with false discovery rate correction. Disease-associated community shifts were quantified using log-ratio indices and co-occurrence network analysis. **Results**: Microbial diversity increased with disease severity, with cervical cancer showing the highest alpha diversity and distinct community composition. Normal samples were uniformly dominated by *Lactobacillus*, whereas LSIL and HSIL exhibited transitional communities with partial loss of lactobacillar dominance and increasing representation of anaerobic taxa. Cervical cancer was associated with depletion of *Lactobacillus* and expansion of anaerobic consortia. A *Lactobacillus*-to-anaerobe log-ratio declined monotonically with disease severity and robustly discriminated invasive cancer from precursor states. Microbial co-occurrence networks became progressively more structured with disease severity, transitioning to dense anaerobic networks in cervical cancer. **Conclusions**: Cervicovaginal microbiome signatures reflect cervical disease stage and may complement existing screening and risk stratification strategies.

## 1. Introduction

Cervical cancer remains a major global health burden, ranking among the leading causes of cancer-related morbidity and mortality in women worldwide [1,2]. Persistent infection with high-risk human papillomavirus (HR-HPV) is a necessary etiological factor for cervical carcinogenesis, but only a minority of infected women progress from transient infection to cervical intraepithelial neoplasia (CIN) and invasive carcinoma [3,4]. This disparity highlights the importance of host, environmental, and microbial cofactors that may modulate HPV persistence, immune responses, and lesion progression [5].

The cervicovaginal microbiome has emerged as a critical determinant of cervical health. In healthy reproductive-age women, this ecosystem is typically dominated by *Lactobacillus* species, which contribute to epithelial barrier integrity, immune modulation, and suppression of pathogenic microorganisms through lactic acid production and maintenance of low vaginal pH [6,7,8,9,10].

On the other hand, disruption of *Lactobacillus* dominance and overgrowth of anaerobic and facultative anaerobic bacteria have been consistently associated with bacterial vaginosis, chronic inflammation, HPV persistence, and increased susceptibility to cervical neoplasia [11,12]. Increasing evidence suggests that cervical disease progression is accompanied by community-wide ecological restructuring involving changes in diversity, composition, and microbial interactions [13].

Previous studies have reported higher microbial richness and diversity in women with CIN and cervical cancer compared with healthy controls, along with distinct beta-diversity profiles that differentiate disease states [14,15,16]. However, findings have been heterogeneous, partly due to differences in study design, population characteristics, sequencing approaches, and analytical methods.

In addition to disease status, host factors have been implicated in shaping the vaginal microbiome [17]. These variables may confound or modify microbiome–disease associations, yet they are often incompletely characterized or inconsistently incorporated into analytical frameworks.

In this study, we aimed to characterize the cervicovaginal microbiome across a spectrum of cervical health states and to identify community-level features that distinguish invasive disease from precursor states.

## 2. Materials and Methods

We conducted a prospective cross-sectional study that included patients who underwent both cervical screening and histopathologic evaluation at Cuza voda Clinical Hospital of Obstetrics and Gynecology, Iasi, Romania, between September 2024 and September 2025. Additional tests (vaginal microbiome profiling) for cervical dysplasia were performed.

The study was conducted in accordance with the Declaration of Helsinki. The protocol was approved by the local institutional ethics committees (Cuza voda Clinical Hospital of Obstetrics and Gynecology—11630/6 September 2024; Grigore T. Popa University of Medicine and Pharmacy Iasi—480/21 October 2024), and written informed consent was obtained from all participants involved in the study.

Only samples with concordant cytologic and histopathologic results were included in the analysis. We also included patients with available results for HPV genotyping, who gave their informed consent for participation in the study. Patients were excluded from the study if any of the following applied: absence of a histopathologic diagnosis, incomplete screening test results, prior treatment for cervical intraepithelial neoplasia or cervical cancer, or insufficient clinical data for the variables of interest.

The following clinically relevant data was retrieved from their medical files: age (years), body mass index (BMI, kg/m^2^), number of pregnancies, place of residence, history of HPV infection, history of sexually transmitted infections (STIs), smoking, alcohol consumption, hormonal contraceptive use, immunosuppression, HPV vaccination status, and HR-HPV positivity.

The groups were segregated based on histopathological diagnoses:

- Normal (Negative for intraepithelial neoplasia, NILM);
- LSIL (low-grade squamous intraepithelial lesion)/(CIN1, cervical intraepithelial neoplasia grade 1);
- HSIL (high-grade squamous intraepithelial lesion)/(CIN2–CIN3, cervical intraepithelial neoplasia grades 2 and 3);
- CCU (cervical carcinoma).

The final dataset included 86 samples: Normal (*n* = 26 patients), LSIL (*n* = 25 patients), HSIL (*n* = 25 patients), and CCU (*n* = 10). A cervix brush (Hologic, Bedford, MA, USA) was used to collect cervical samples for the Pap test and HPV genotyping. The ThinPrep liquid-based procedure was used to prepare the samples in accordance with the manufacturer’s instructions (ThinPrep-Hologic, Bedford, MA, USA). All samples were subjected to human papillomavirus detection and genotyping using AllplexTM HPV28 Detection (Seegene Technologies Inc. Europe, Dusseldorf, Germany) in accordance with the manufacturer’s instructions.

Vaginal samples for microbiota analysis were collected using the OMNIgene^®^•VAGINAL collection device (DNA Genotek (Stittsville, ON, Canada)). All samples were collected according to the manufacturer’s instructions and processed uniformly.

Microbial DNA was extracted following standardized protocols recommended by the manufacturer. Library preparation for bacterial profiling was performed using the Oxford Nanopore Technologies (ONT) (Oxford, UK) 16S Barcoding Kit 1–24 (SQK-16S024), which targets the full-length 16S rRNA gene and enables multiplexing of up to 24 samples per sequencing run.

Sequencing libraries were loaded onto R9.4.1 flow cells (FLO-MIN106) and sequenced on the ONT MinION platform. Flow cell priming, library loading, and sequencing were performed following ONT manufacturer recommendations.

Taxonomic classification was performed using 16S rRNA gene-based pipelines, yielding genus-level bacterial profiles for each sample. Only taxa consistently detected above background levels were retained for downstream analyses.

All wet-lab procedures were performed strictly according to the manufacturer’s protocols for the OMNIgene^®^•VAGINAL device, ONT 16S Barcoding Kit 1–24, and MinION sequencing platform.

Alpha diversity was assessed using observed genus richness and the Shannon diversity index, calculated on relative abundance data. Differences across diagnostic groups were evaluated using the Kruskal–Wallis test, followed by pairwise Mann–Whitney U tests with Benjamini–Hochberg false discovery rate (FDR) correction. Effect sizes were quantified using Cliff’s delta, and effect magnitude was interpreted using established thresholds (negligible, small, medium, large).

Beta diversity was quantified using the Aitchison distance, computed as Euclidean distance in CLR-transformed space. Ordination was performed using principal coordinates analysis (PCoA). Global differences in community composition across diagnostic categories were tested using PERMANOVA with 9999 permutations. Pairwise PERMANOVA comparisons were performed between diagnostic groups, with FDR correction applied to *p* values. To assess whether observed compositional differences were influenced by heterogeneity in within-group variance, PERMDISP was conducted using the same distance matrix.

Associations between vaginal microbiome composition and host-related variables (including physical activity, HPV vaccination status, menstrual cycle phase, and additional clinical and behavioral factors) were evaluated using PERMANOVA based on Aitchison distances. Each factor was tested independently using 9999 permutations.

Differential abundance testing was performed using ANCOM-BC2, a bias-corrected compositional method that estimates log-fold changes while accounting for sampling variability and compositional constraints. Global tests and biologically relevant pairwise contrasts were conducted across diagnostic categories. Genera with an FDR-adjusted q value ≤ 0.10 were considered statistically significant. Log-fold change estimates and 95% confidence intervals were visualized using forest plots.

To quantify disease-associated shifts in community structure, two log-ratio indices were computed using CLR-transformed data. The first was a predefined *Lactobacillus*-to-anaerobe log-ratio, contrasting *Lactobacillus* against a curated set of obligate anaerobic genera (including *Gardnerella*, *Prevotella*, *Dialister*, and related taxa), representing the ecological transition from *Lactobacillus*-dominated to dysbiotic states. The second was a data-driven composite log-ratio, constructed by contrasting geometric means of genera consistently increased versus decreased in CCU relative to Normal samples.

Log-ratio values were compared across diagnostic groups using Kruskal–Wallis tests followed by pairwise Mann–Whitney U tests with FDR correction. Effect sizes were quantified using Cliff’s delta. Diagnostic performance was evaluated using receiver operating characteristic (ROC) curves.

Genus–genus associations were estimated using Spearman rank correlations computed on CLR-transformed abundances. Correlations involving constant vectors or undefined coefficients were excluded.

Microbial co-occurrence networks were inferred separately for each diagnostic group using samples with available histopathological diagnoses. To ensure network stability and reduce spurious associations, taxa were filtered within each group prior to network construction. Genera were retained if they met the following criteria:

- Prevalence ≥ 30% of samples within the group (≥ 40% for CCU due to smaller sample size);
- Non-zero variance across samples.

From the genera passing these criteria, a maximum of 20 genera with the highest total abundance within each group were selected.

Pairwise Spearman correlations were computed on CLR-transformed data. *p* values were adjusted using the Benjamini–Hochberg FDR procedure. Edges were retained if they met both statistical and strength criteria:

- |ρ| ≥ 0.60 and q < 0.05 for Normal, LSIL, and HSIL groups;
- |ρ| ≥ 0.70 and q < 0.05 for CCU.

Undirected weighted networks were constructed using NetworkX 3.6.1, with nodes representing genera and edges weighted by Spearman’s ρ.

Network structure was characterized using standard graph metrics, including the following:

- Number of nodes (genera retained);
- Number of edges (significant correlations);
- Network density;
- Number of connected components.

All analyses were conducted in Python 3.12 and Stata 19.5 (StataCorp LLC, College Station, TX, USA). A *p* value < 0.05 was considered statistically significant.

## 3. Results

### 3.1. Baseline Characteristics of the Included Patients

The final cohort included 86 patients, and their clinical characteristics are presented in Table 1. Women diagnosed with LSIL and HSIL were younger on average (35.40 ± 8.86 years and 37.56 ± 9.88 years, respectively) compared with those in the normal group (42.35 ± 9.40 years, *p* = 0.0109). The highest mean age was observed in the CCU group (45.10 ± 8.80 years), suggesting a trend toward increasing age with disease severity and progression from precursor lesions to invasive cervical cancer.

HR-HPV positivity increased progressively with lesion severity, being present in 46.15% of women with normal cytology, 88.00% of those with LSIL, and 100% of women with HSIL and CCU (*p* < 0.001). Vaccination coverage was highest among women with LSIL (40.00%) and HSIL (28.00%), while no vaccinated individuals were identified in the CCU group (*p* = 0.049).

### 3.2. Microbial Diversity Association with Lesion Severity

**Figure 1:**
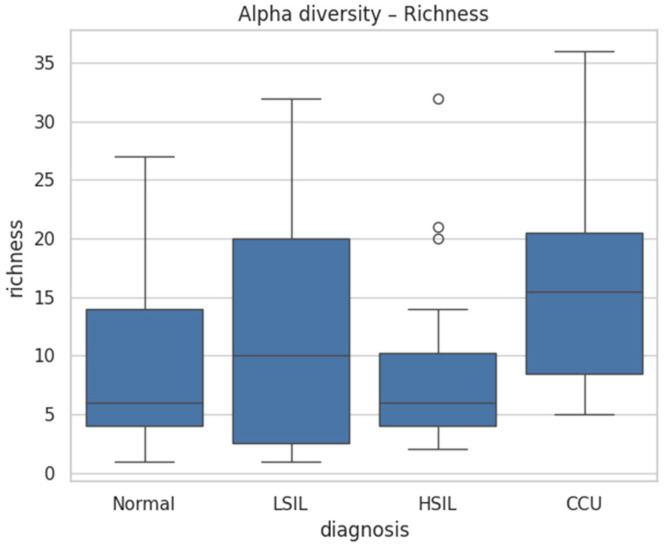
Observed richness.

**Figure 2:**
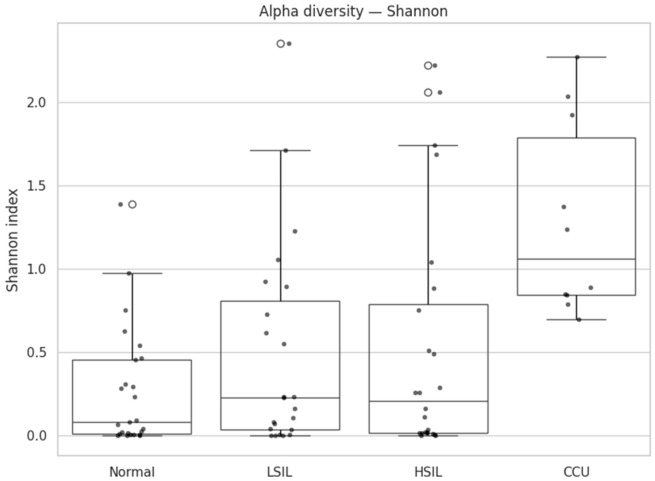
Shannon diversity.

Cervicovaginal microbial communities exhibited a progressive restructuring across the spectrum of cervical disease. Normal samples displayed low richness (median 6.0, Q1–Q3 4.0–14.0) and Shannon diversity (median 0.08, Q1–Q3 0.01–0.45), reflecting low-complexity ecosystems (Table 2; Figure 1 and Figure 2).

LSIL and HSIL samples showed intermediate diversity (LSIL richness 10.0 [2.5–20.0], Shannon 0.23 [0.03–0.81]; HSIL richness 6.0 [4.0–10.3], Shannon 0.21 [0.01–0.78]), indicating partial destabilization of these communities. Cervical cancer samples exhibited markedly higher richness (15.5 [8.5–20.5]) and Shannon index (1.06 [0.84–1.78]) (Table 2; Figure 1 and Figure 2).

**Table 2:** Alpha diversity metrics across diagnostic groups.

Diagnosis	Richness (Median)	Richness (Q1–Q3)	Shannon Index (Median)	Shannon Index (Q1–Q3)
Normal	6.0	4.0–14.0	0.08	0.01–0.45
LSIL	10.0	2.5–20.0	0.23	0.03–0.81
HSIL	6.0	4.0–10.3	0.21	0.01–0.78
CCU	15.5	8.5–20.5	1.06	0.84–1.78

CCU, cervical cancer (invasive carcinoma); HSIL, high-grade squamous intraepithelial lesion; LSIL, low-grade squamous intraepithelial lesion .

Pairwise comparisons revealed that cervical cancer samples presented significantly higher Shannon diversity and richness than all non-cancer groups. Specifically, cervical cancer samples versus normal samples showed a Shannon index difference with FDR-adjusted *p* = 0.000028 and a large effect size (Cliff’s delta = −0.90), and richness was also significantly higher (FDR-adjusted *p* = 0.0471, Cliff’s delta = −0.53) (Table 3 and Table 4). Compared with LSIL, cervical cancer samples exhibited elevated Shannon diversity (FDR-adjusted *p* = 0.0068, Cliff’s delta = −0.67) and a smaller increase in richness (FDR-adjusted *p* = 0.427, Cliff’s delta = −0.21). Relative to HSIL, cervical cancer samples displayed higher Shannon diversity (FDR-adjusted *p* = 0.0068, Cliff’s delta = −0.65) and richness (FDR-adjusted *p* = 0.0471, Cliff’s delta = −0.54).

On the other hand, differences among non-cancer samples were modest or negligible: normal versus LSIL (Shannon FDR = 0.375, Cliff’s delta = −0.18; richness FDR = 0.427, delta = −0.19), normal versus HSIL (Shannon FDR = 0.375, delta = −0.17; richness FDR = 0.740, delta = −0.06), and LSIL versus HSIL (Shannon FDR = 0.992, delta = −0.00; richness FDR = 0.427, delta = 0.17).

### 3.3. Community Composition Differs by Disease Severity

Multivariate analyses revealed that cervicovaginal microbial community composition diverges progressively across cervical disease severity. Global PERMANOVA based on Aitchison distances confirmed a strong effect of diagnosis on microbiome structure (pseudo-F = 2.43, *p* = 0.0006; 9999 permutations; *n* = 82) (Table 5), indicating that diagnostic category accounts for a significant proportion of compositional variance.

Pairwise PERMANOVA comparisons highlighted that the largest shifts in community structure were associated with cervical cancer samples. Specifically, normal versus cervical cancer samples exhibited the strongest separation (FDR-adjusted *p* = 0.0006), followed by significant differences between HSIL and cervical cancer samples (FDR-adjusted *p* = 0.0296) and LSIL and cervical cancer samples (FDR-adjusted *p* = 0.0377) (Table 6).

In contrast, differences between intermediate lesion stages were smaller and, in some cases, not statistically significant, such as LSIL versus HSIL (FDR-adjusted *p* = 0.2228).

Normal samples also differed significantly from HSIL (FDR-adjusted *p* = 0.0138), indicating that compositional changes begin early but intensify as lesions progress (Table 6).

**Figure 3:**
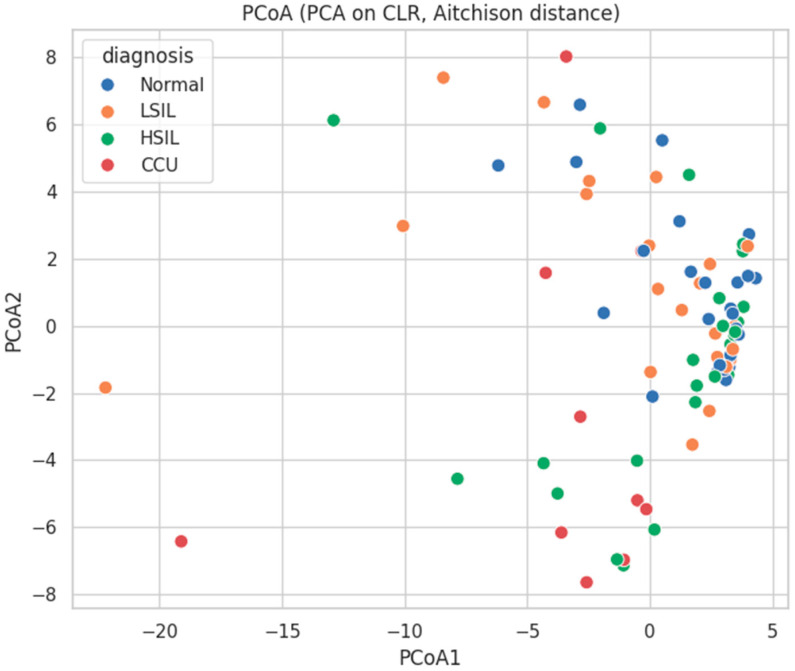
Principal Coordinates Analysis (PCoA) of Vaginal Microbiome Composition.

The PCoA scatter plot (Figure 3) visualizes these compositional differences. Samples cluster broadly by diagnostic category, with normal and LSIL samples forming a relatively tight cluster near the origin, HSIL samples slightly more dispersed, and cervical cancer samples spreading further along both PCoA1 and PCoA2 axes, reflecting their higher dissimilarity.

Table 7 presents the mean Aitchison distances between vaginal microbiome profiles across diagnostic categories. The largest distances were observed between cervical cancer samples and all other groups (Normal: 14.98, LSIL: 15.98, HSIL: 14.88), indicating that cervical cancer microbiomes are highly divergent from non-cancer microbiomes.

On the other hand, the distances among non-cancer groups were smaller (Normal–LSIL: 10.74, Normal–HSIL: 9.91, LSIL–HSIL: 11.83), suggesting more reduced compositional changes between intermediate lesion stages.

### 3.4. Increasing Within-Group Heterogeneity with Progression

To determine whether variation in within-group dispersion influenced these patterns, PERMDISP analyses were performed. Dispersion differed significantly across diagnostic categories (F = 4.97, *p* = 0.0034), with mean dispersions increasing progressively from Normal (5.86) to LSIL (8.69), HSIL (7.35), and cervical cancer samples (11.42), suggesting that microbial communities become increasingly heterogeneous with lesion severity (Table 8).

**Figure 4:**
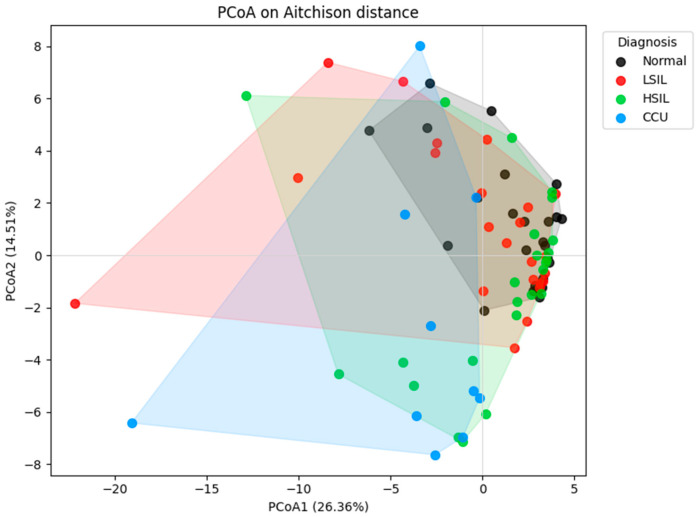
PCoA with Convex Hulls Depicting Vaginal Microbiome Community Structure.

The convex hull PCoA plot (Figure 4) further illustrates group dispersion. The hulls for normal, LSIL, and HSIL largely overlap, reflecting their moderate similarity, while the convex hull for cervical cancer samples extends away from other groups, confirming both their compositional divergence and higher intra-group variability.

### 3.5. Host Factors Associated with Microbiome Composition

Table 9 summarizes PERMANOVA analyses assessing the association between host factors and vaginal microbiome composition. Among the factors tested, only a few were significantly associated with microbiome variation. Physical activity showed a significant effect (pseudo-F = 1.836, *p* = 0.007), suggesting that activity levels influence overall community structure. HPV vaccination status also had a significant effect (pseudo-F = 2.594, *p* = 0.014), indicating that vaccinated and unvaccinated individuals harbor distinct microbial communities. Additionally, current menstrual cycle phase was modestly significant (pseudo-F = 1.670, *p* = 0.046), implying that hormonal fluctuations may contribute to microbiome variation.

### 3.6. Loss of Lactobacillus Dominance and Anaerobe Enrichment Across Lesion Severity

**Figure 5:**
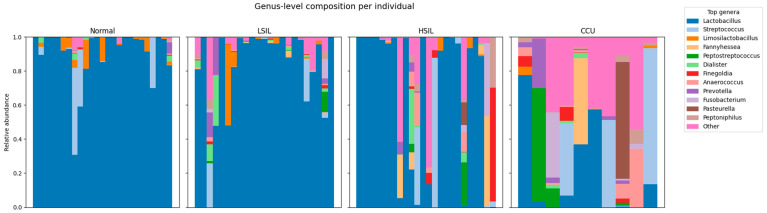
Genus level composition of vaginal microbiome per individual.

**Figure 6:**
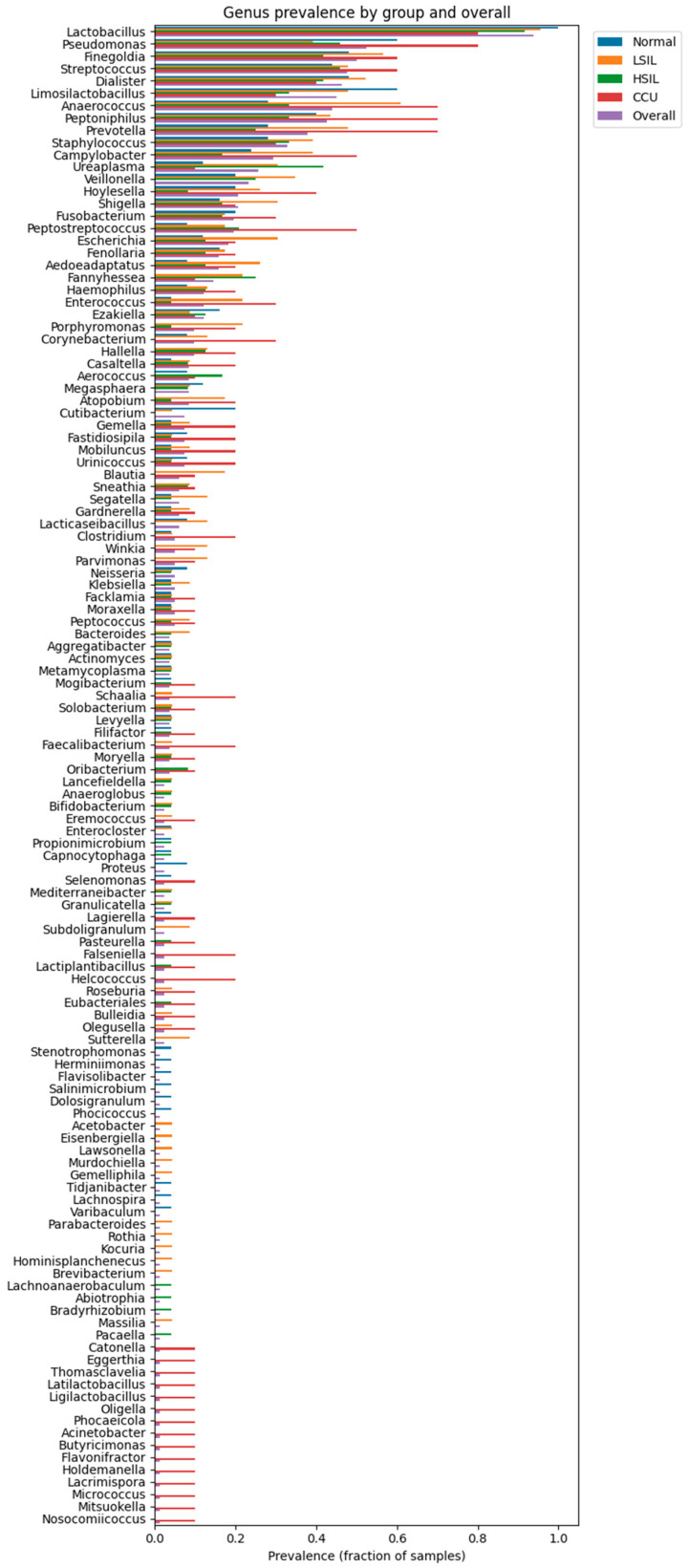
Genus’s prevalence per groups and overall.

Individual-level stacked bar plots revealed marked shifts in the taxonomic structure of the cervicovaginal microbiome across diagnostic categories (Figure 5 and Figure 6). Normal samples were uniformly dominated by *Lactobacillus*, with only minor contributions from other genera. In LSIL and HSIL, this structure became progressively more heterogeneous, with increasing representation of anaerobic taxa such as *Prevotella*, *Peptostreptococcus*, *Dialister*, and *Anaerococcus*. Cervical cancer samples exhibited the most significant restructuring: *Lactobacillus* dominance was lost in nearly all patients and replaced by highly diverse, polymicrobial communities enriched in inflammatory anaerobes (*Prevotella*, *Peptoniphilus*, *Fannyhessea*, *Finegoldia*, *Fusobacterium*).

Normal samples were dominated by *Lactobacillus*, which accounted for a median relative abundance of 98.77% and was detected in 100% of samples. Although *Lactobacillus* prevalence remained high in LSIL (median 96.33%, prevalence 95.65%) and HSIL (median 96.08%, prevalence 91.67%), its dominance progressively weakened, as reflected by declining mean relative abundance (LSIL 83.48%, HSIL 66.88%) and increasing representation of non-lactobacillar taxa.

In contrast, cervical cancer samples exhibited a marked loss of *Lactobacillus* dominance, with a median relative abundance of only 5.02% despite persistence in 80% of samples (Table 10). This loss of *Lactobacillus* was accompanied by increased abundance and prevalence of anaerobic and facultative anaerobic genera. *Prevotella* (median 1.39%, prevalence 70%), *Anaerococcus* (median 0.88%, prevalence 70%), *Peptoniphilus* (median 0.66%, prevalence 70%), *Fannyhessea* (median 0.00%, prevalence 10%), *Finegoldia* (median 0.07%, prevalence 60%), and *Fusobacterium* (median 0.00%, prevalence 30%) were substantially enriched in cervical cancer samples, showing both higher mean relative abundance and increased prevalence compared with non-cancer groups. Several of these genera were present at low median abundance but high prevalence, indicating widespread low-level colonization rather than dominance by single taxa (Table 10).

Intermediate lesion categories (LSIL and HSIL) displayed transitional microbial profiles, characterized by partial retention of lactobacillar dominance alongside increased prevalence of anaerobic genera. For example, *Anaerococcus* prevalence increased from 28% in normal samples to 60.87% in LSIL and 33.33% in HSIL, while *Dialister* prevalence rose from 48% in normal samples to 52.17% in LSIL. Similarly, *Peptoniphilus* prevalence increased from 40% in normal samples to 43.48% in LSIL and 33.33% in HSIL, consistent with a gradual ecological shift rather than an abrupt compositional change (Table 10).

### 3.7. Differential Abundance and Lactobacillus-to-Anaerobe Compositional Shifts Across Cervical Disease Severity

Global and pairwise analyses using ANCOM-BC2 (Table 11) corroborated the predominant role of *Lactobacillus* in driving microbial differences across diagnostic categories. The global test indicated a trend for *Lactobacillus* depletion across disease stages (*p* = 0.0038, FDR q = 0.369), although this did not reach statistical significance after correction for multiple testing. Pairwise comparisons further demonstrated consistent reductions in *Lactobacillus* abundance with disease severity: logFC = −5.47 in cervical cancer versus normal, −4.49 in cervical cancer versus LSIL, −1.38 in HSIL versus LSIL, and −0.98 in LSIL versus normal. *Prevotella* exhibited increased abundance in cervical cancer versus normal (logFC = 2.76, q = 0.160) and versus LSIL (logFC = 1.47, q = 0.929), but decreased modestly in HSIL versus LSIL (logFC = −1.22, q = 0.422).

*Dialister* and *Staphylococcus* showed more subtle and inconsistent patterns across diagnostic categories. For *Dialister*, the log fold changes were −0.24 for cervical cancer versus normal (q = 0.409), −0.98 for cervical cancer versus LSIL (q = 0.929), −1.17 for HSIL versus LSIL (q = 0.422), and 0.74 for LSIL versus normal (q = 0.657). *Staphylococcus* exhibited logFC values of −0.26 in cervical cancer versus normal (q = 0.391), −0.85 in cervical cancer versus LSIL (q = 0.929), −0.82 in HSIL versus LSIL (q = 0.479), and 0.58 in LSIL versus normal (q = 0.657). These data indicate that, unlike *Lactobacillus*, both *Dialister* and *Staphylococcus* show minor, non-significant fluctuations across lesion severity without consistent directional trends.

**Figure 7:**
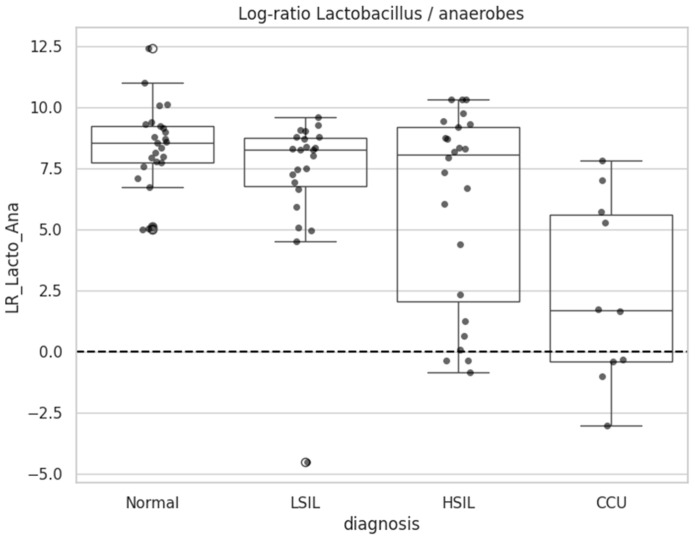
Log-ratio of *Lactobacillus* to anaerobic bacteria across diagnostic groups.

To capture community-wide compositional shifts, log-ratio analyses were performed. A predefined ratio contrasting *Lactobacillus* against a panel of anaerobic genera showed a strong monotonic decline, reaching its lowest values in cervical cancer samples (Figure 7).

Analysis of the log-ratio contrasting *Lactobacillus* abundance against anaerobic genera revealed significant differences across cervical disease categories (Kruskal–Wallis H = 18.69, *p* = 0.0003; Table 12). Normal samples exhibited high median log-ratio values (5.06, Q1–Q3: 4.41–6.36), consistent with strong *Lactobacillus* dominance and low relative abundance of anaerobes.

Intermediate lesions displayed partially destabilized communities, with LSIL samples showing a median log-ratio of 3.57 (Q1–Q3: 3.05–5.22) and HSIL samples a median of 4.34 (Q1–Q3: 0.21–5.07), reflecting heterogeneous microbial states. Cervical cancer samples demonstrated markedly reduced log-ratio values (median 0.51, Q1–Q3: −0.63–1.26), indicating a pronounced shift toward anaerobe-rich microbiomes and loss of *Lactobacillus* dominance.

Pairwise comparisons (Table 13) confirmed that the largest differences were observed in comparisons involving cervical cancer. Log-ratio values differed significantly between Normal and cervical cancer (*p* = 8.7 × 10^−5^, FDR q = 5.2 × 10^−4^) and between LSIL and cervical cancer (*p* = 0.0040, FDR q = 0.0120), both with large effect sizes (Cliff’s delta = 0.86 and 0.64, respectively). Differences between Normal and LSIL (*p* = 0.0149, q = 0.0223) and Normal and HSIL (*p* = 0.0135, q = 0.0223) were of medium magnitude (Cliff’s delta = 0.41). No meaningful difference was detected between LSIL and HSIL (*p* = 0.710, q = 0.710, Cliff’s delta = 0.07), indicating that early lesion categories exhibit only partial shifts in microbial composition. Comparisons of HSIL versus cervical cancer showed a medium effect size (Cliff’s delta = 0.40) but did not reach statistical significance after FDR correction (q = 0.0871).

To ensure consistency with non-parametric approaches, the same log-ratio was evaluated using Mann–Whitney tests (Table 14). Significant differences were observed primarily in comparisons involving cervical cancer, including normal versus cervical cancer (*p* = 0.00010, q = 0.00060) and LSIL versus cervical cancer (*p* = 0.00107, q = 0.00643). Differences between HSIL and cervical cancer were nominally significant (*p* = 0.0120, q = 0.0717), whereas comparisons among non-cancer categories (Normal vs. LSIL, Normal vs. HSIL, LSIL vs. HSIL) were non-significant after FDR adjustment.

### 3.8. Co-Occurrence Patterns Within Diagnostic Groups

**Figure 8:**
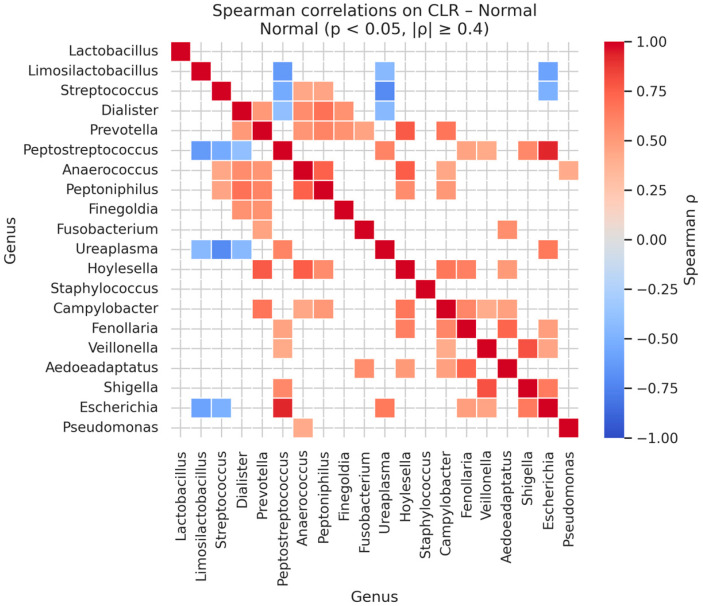
Significant Genus-Level Spearman Correlations in CLR-Transformed Normal Microbiomes.

Analysis of genus–genus co-occurrence patterns within each diagnostic category revealed strong, statistically significant correlations among specific bacterial taxa (Table 15 and Figure 8). In normal samples, the strongest positive correlations were observed between *Peptostreptococcus* and *Escherichia* (Spearman’s ρ = 0.92, *p* < 0.001), *Veillonella* and *Shigella* (ρ = 0.80, *p* < 0.001), and *Prevotella* with *Hoylesella* (ρ = 0.77, *p* < 0.001).

Additional notable associations included *Anaerococcus* with *Hoylesella* (ρ = 0.76, *p* < 0.001) and with *Peptoniphilus* (ρ = 0.74, *p* < 0.001), reflecting coordinated presence of anaerobic and facultative anaerobic taxa within the healthy cervicovaginal microbiome.

**Figure 9:**
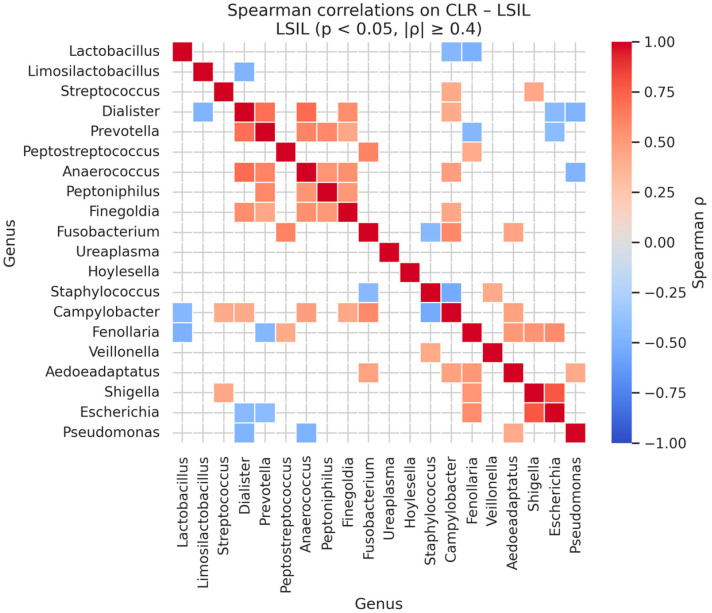
Significant Genus-Level Spearman Correlations in CLR-Transformed LSIL Microbiomes.

In LSIL samples, strong correlations persisted among facultative anaerobes and anaerobic genera, including *Shigella* and *Escherichia* (ρ = 0.78, *p* < 0.001), *Dialister* with *Anaerococcus* (ρ = 0.71, *p* < 0.001) and with *Prevotella* (ρ = 0.69, *p* < 0.001), as well as *Peptostreptococcus* with *Fusobacterium* (ρ = 0.60, *p* < 0.01) and *Prevotella* with *Anaerococcus* (ρ = 0.60, *p* < 0.01). These results indicate that early lesion stages are characterized by moderate co-occurrence among anaerobic and facultative anaerobic taxa, consistent with partial destabilization of the microbiome (Table 15 and Figure 9).

**Figure 10:**
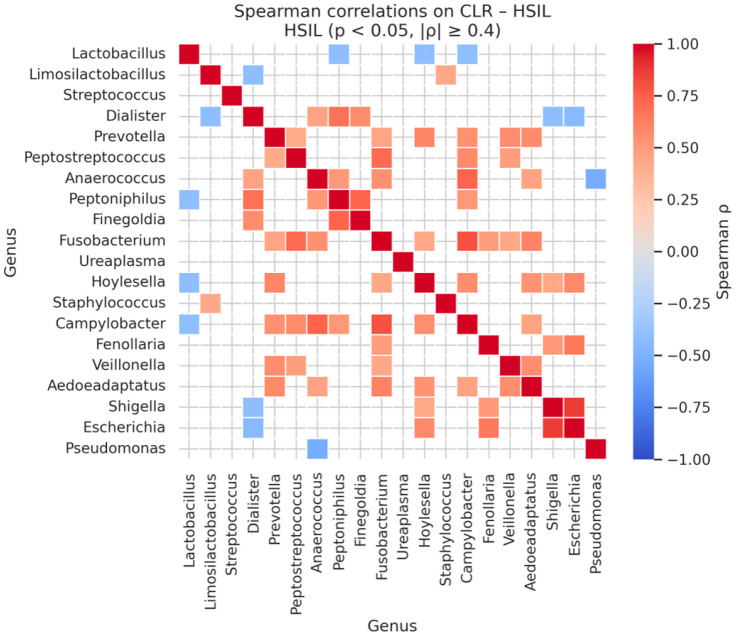
Significant Genus-Level Spearman Correlations in CLR-Transformed HSIL Microbiomes.

In HSIL samples, correlations were generally stronger, with *Shigella* and *Escherichia* remaining highly correlated (ρ = 0.87, *p* < 0.001). Other strong associations included *Fusobacterium* with *Campylobacter* (ρ = 0.80, *p* < 0.001), *Peptoniphilus* with *Finegoldia* (ρ = 0.74, *p* < 0.001), and *Anaerococcus* with *Campylobacter* (ρ = 0.74, *p* < 0.001), reflecting emerging co-occurrence networks among pathogenic anaerobes as lesion severity increases (Table 15 and Figure 10).

**Figure 11:**
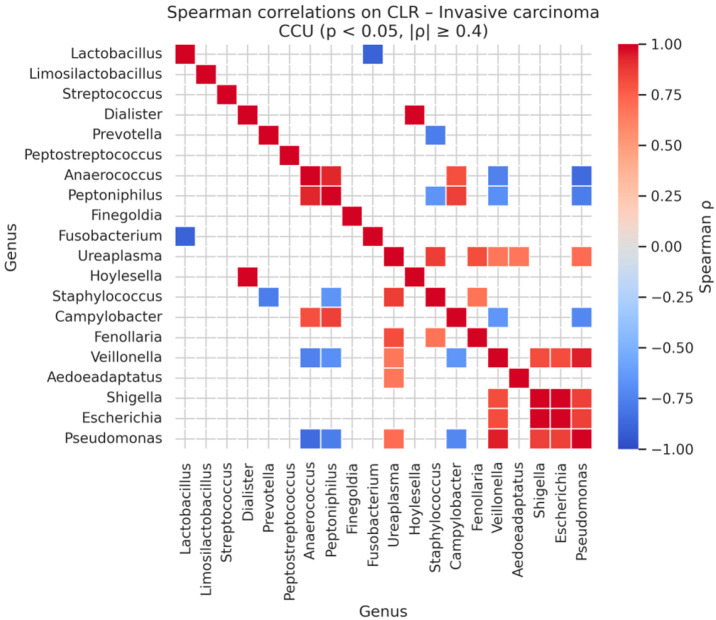
Significant Genus-Level Spearman Correlations in CLR-Transformed Cervical Cancer Microbiomes.

In cervical cancer samples, genus–genus correlations reached the highest magnitudes. Perfect or near-perfect correlations were observed between *Shigella* and *Escherichia* (ρ = 1.00, *p* < 0.001) and *Dialister* with *Hoylesella* (ρ = 0.99, *p* < 0.001). Other strong associations included *Veillonella* with *Pseudomonas* (ρ = 0.94, *p* < 0.001), *Anaerococcus* with *Peptoniphilus* (ρ = 0.93, *p* < 0.001), and *Ureaplasma* with *Staphylococcus* (ρ = 0.87, *p* < 0.01). These results indicate that advanced disease is associated with highly structured co-occurrence networks among anaerobic and facultative anaerobic genera, reflecting a more deterministic, polymicrobial community state in invasive carcinoma (Table 15 and Figure 11).

Correlation network analysis revealed marked differences in microbial community structure across disease stages (Table 16). In the normal group, the network was sparse, with only seven genera retained after prevalence filtering and a single significant association detected between *Dialister* and *Peptoniphilus* (Spearman’s ρ = 0.686, q = 0.0032). The low network density (0.0476) and high number of disconnected components (six components) indicated a weakly interacting microbial community, consistent with a stable ecosystem.

In contrast, the LSIL group exhibited a substantial increase in network complexity, with fifteen genera retained and three strong positive associations. Notably, *Dialister* emerged as a central node, showing significant correlations with both *Anaerococcus* (ρ = 0.708, q = 0.0084) and *Prevotella* (ρ = 0.689, q = 0.0098), while a strong association between *Escherichia* and *Shigella* (ρ = 0.776, q = 0.0014) indicated coordinated expansion of facultative pathobionts. Despite this increased connectivity, the LSIL network remained fragmented, with a low density (0.0286) and a high number of disconnected components (12 components), suggesting heterogeneous microbial configurations characteristic of an early dysbiotic transition rather than a fully consolidated community state.

The HSIL group displayed an intermediate network structure, characterized by ten retained genera and two significant edges, representing a reduction in both node and edge counts relative to LSIL. However, the remaining associations were strong and predominantly involved anaerobic taxa, including *Finegoldia–Peptoniphilus* (ρ = 0.740, q = 0.0016) and *Dialister–Peptoniphilus* (ρ = 0.687, q = 0.0047). Network density (0.0444) suggested consolidation around a smaller number of tightly co-occurring anaerobic consortia, potentially reflecting increasing environmental constraints associated with lesion progression.

The cervical cancer samples showed the highest degree of network connectivity despite the smallest sample size, with eleven genera retained and four significant edges. This group exhibited the highest network density (0.0727) across all diagnostic categories. Strong positive correlations were observed among several anaerobic genera, including *Dialister–Hoylesella* (ρ = 0.988, q = 5.1 × 10^−6^) and *Anaerococcus–Peptoniphilus* (ρ = 0.927, q = 0.0031). In addition, a strong negative association between *Anaerococcus* and *Pseudomonas* (ρ = −0.855, q = 0.0225) indicated competitive exclusion within the cancer-associated microbiome.

## 4. Discussion

In this study, we comprehensively characterized the dynamics of the cervicovaginal microbiome across the spectrum of cervical disease severity, from normal cytology to intraepithelial lesions and invasive cervical carcinoma. Our results revealed a progressive, coherent, and multifaceted restructuring of the microbial community that is significantly associated with disease progression, loss of *Lactobacillus* dominance, and the emergence of complex polymicrobial anaerobic communities.

A central finding of our analyses is that the most pronounced differences in microbial diversity and composition are concentrated in cervical cancer, whereas transitions among non-cancer states (Normal, LSIL, HSIL) are comparatively subtle. Both alpha diversity metrics (richness and Shannon index) and multivariate analyses (PERMANOVA, PCoA, Aitchison distances) consistently demonstrate a clear ecological break between cancer and all other diagnostic categories. This pattern suggests that severe dysbiosis is not an early event in cervical disease but rather a defining feature of invasive carcinoma, likely reflecting profound alterations in the local cervical microenvironment accompanying malignant transformation.

Quantitative analyses across multiple studies consistently showed that HPV infection, cervical intraepithelial neoplasia, and cervical cancer were associated with increased microbial richness and higher Shannon diversity, together with distinct beta-diversity patterns. A large meta-analysis of 507 cervical samples showed significantly higher Shannon diversity and evenness in CIN and cervical cancer compared with normal controls, with a clear increasing trend across normal controls, HPV infection, CIN, and cancer, although differences between CIN and cancer were not significant [18]. The same meta-analysis showed that cervical cancer was characterized by enrichment of opportunistic pathogenic taxa, including *Streptococcus*, *Fusobacterium*, *Pseudomonas*, and *Anaerococcus*, alongside a marked depletion of *Lactobacillus* compared with normal controls. On the other hand, the CIN group exhibited significantly increased relative abundances of *Gardnerella*, *Sneathia*, *Pseudomonas*, and *Fannyhessea* relative to other bacterial taxa [18].

Consistently, a cross-sectional study that included a large HPV-positive cohort (*n* = 692 patients) demonstrated significantly greater diversity in high-grade CIN compared with lower-grade lesions using Shannon-based indices [19]. The authors also showed that high-grade CIN was associated with coordinated downregulation of multiple metabolic and regulatory pathways, including the phosphotransferase system, transcription-related functions, fructose and mannose metabolism, amino sugar and nucleotide sugar metabolism, and galactose metabolism. Also, CIN was characterized by a distinct vaginal microbiome configuration marked by depletion of *Lactobacillus* and *Pseudomonas* and concomitant enrichment of *Gardnerella*, *Prevotella*, and *Dialister* [19].

Similarly, another cohort of HPV-positive patients reported an increase in mean Shannon diversity from 1.06 in HPV-negative patients to 2.23 in HPV-positive patients (*p* = 0.002), with values rising across normal cytology, CIN, and cancer, even though not all histology-specific comparisons reached statistical significance. Moreover, HPV-negative normal samples clustered distinctly from CIN and cancer cases in ordination space, indicating fundamentally different community structures [20]. A longitudinal CIN progression study including controls, LSIL, HSIL, and invasive cervical cancer further confirmed increasing Shannon and Simpson diversity with lesion severity in parallel with progressive loss of *Lactobacillus* dominance [21].

A systematic review and meta-analysis reinforced these findings, reporting significantly higher richness and Shannon diversity in vaginal samples from cervical cancer cases compared with controls, as well as higher Shannon diversity in cervical samples, although richness measures were less consistent across sample types [22].

Beta-diversity and multivariate analyses further supported the presence of disease-associated shifts in overall community composition [18]. Recent literature data incorporating compositionality-aware methods, such as Aitchison distances and robust compositional models, largely confirmed significant class separation after adjusting for study-specific effects, further strengthening evidence for consistent, disease-associated restructuring of the cervicovaginal microbiome [23,24].

Contrary to a model of gradual, linear microbial deterioration across lesion stages, our data indicate that LSIL and HSIL represent intermediate and unstable states characterized by increased heterogeneity rather than uniform shifts in diversity or community structure. This is supported by the small or negligible effect sizes observed in pairwise comparisons among Normal, LSIL, and HSIL groups for both alpha diversity and overall composition. Thus, microbiome alterations appear to accumulate gradually but manifest abruptly upon transition to invasive cancer.

The marked increase in alpha diversity observed in cervical cancer, together with higher within-group dispersion, points to a loss of community-level constraint and the emergence of more permissive and less stable microbial assemblages. Anaerobic growth in cervical cancer most likely results from the interplay of microbial ecology, inflammation, and persistent HPV infection. Immune dysregulation and HPV-induced epithelium disruption decrease colonization resistance, but *Lactobacillus* depletion raises pH and lessens ecological limitations, which promotes anaerobic expansion [25,26]. Consequently, inflammatory metabolites produced by anaerobes may enhance viral persistence and alter the milieu to facilitate the growth of cancer [27,28].

A key feature of disease progression identified in this study is the progressive loss of *Lactobacillus* dominance. While *Lactobacillus* remained prevalent in most non-cancer samples, its relative abundance declined in parallel with increasing lesion severity, accompanied by expansion of anaerobic taxa. Compositional log-ratio analyses, which are robust to the constraints of relative abundance data, revealed a strong monotonic decrease in the *Lactobacillus*-to-anaerobe ratio, with the most pronounced differences involving cervical cancer. These findings support the notion that imbalance between lactic acid-producing bacteria and anaerobic, pro-inflammatory taxa.

Across multiple cohorts, cervicovaginal microbial communities have been consistently classified into *Lactobacillus*-dominated community state types (CSTs I–III/V) and anaerobe-dominated CST IV, revealing systematic proportional shifts with increasing disease severity. In a Chinese cohort spanning normal cytology, HPV infection, LSIL, HSIL, and cervical cancer, *Lactobacillus* remained the most abundant genus overall but declined progressively with lesion severity, while anaerobic taxa, including *Prevotella*, *Anaerococcus*, *Sneathia*, *Megasphaera*, *Fusobacterium*, *Veillonellaceae*, and *Porphyromonas uenonis*, were disproportionately enriched in cancer cases [29]. Normal samples were predominantly classified as CST III (*L. iners*–dominated), whereas HPV infection and subsequent lesion development were associated with a stepwise increase in CST IV prevalence, reflecting a marked reduction in the *Lactobacillus*:anaerobe balance as disease progressed [29].

Similar patterns were observed in a mixed-ethnicity cohort, where the prevalence of high-diversity, *Lactobacillus*-poor CST IV increased from 10% in healthy controls to 40% in invasive cervical cancer, accompanied by declining *Lactobacillus* abundance and increasing representation of strict anaerobes such as *Sneathia*, *Anaerococcus*, and *Peptostreptococcus* [29].

A culturomics-based comparison of non-cancer and cervical cancer samples similarly showed dominance of Firmicutes and lactic acid bacteria in non-cancer samples, contrasted with depletion or complete absence of *Lactobacillus*, increased anaerobic diversity, and frequent isolation of *Bacteroides* and other opportunistic anaerobes in cervical cancer, suggesting that the *Lactobacillus*: anaerobe ratio approaches zero in many affected women [30].

In a longitudinal study of CIN2 patients followed for 24 months, *Lactobacillus*-dominant communities (≥81.6% *Lactobacillus*) were present in 65.5% of women at baseline, whereas those with *Lactobacillus*-depleted, strict-anaerobe–rich communities (<54.2% *Lactobacillus*) exhibited a 3.2- to 3.6-fold increased odds of CIN2 persistence at 12 months (adjusted OR 3.56, 95% CI 1.31–9.60) [31]. These findings highlight the *Lactobacillus*: anaerobe ratio as a biologically meaningful metric that distinguishes regressive from persistent diseases and links microbial community structure to clinical outcomes.

Notably, genus-level differential abundance analyses (ANCOM-BC2) identified consistent directional trends but few statistically significant associations after correction for multiple testing. This highlights an important limitation of single-taxon approaches in highly variable microbial ecosystems and suggests that biologically meaningful changes are better captured at the community level or through compositional contrasts, such as log-ratios and network-based analyses.

Network and correlation analyses further illuminated the reorganization of microbial community structure across disease stages. Normal samples exhibited sparse, weakly connected networks, characteristic of a stable *Lactobacillus*-dominated state with limited inter-taxon interactions. With increasing lesion severity, networks became denser and more structured, culminating in cervical cancer with tightly interconnected anaerobic consortia. The presence of strong negative correlations in cancer samples further suggests competitive interactions and niche exclusion, hallmarks of perturbed and highly constrained microbial systems.

Among host-related factors, only physical activity, HPV vaccination status, and menstrual cycle phase were significantly associated with overall microbiome composition. However, evidence on physical activity, HPV vaccination, and menstrual cycle phase as modifiers of the vaginal microbiota specifically in cervical cancer is very sparse. Literature data suggested that vaginal microbial community structure is strongly influenced by hormonal fluctuations and becomes less stable and more diverse during menstruation, whereas pregnancy is associated with greater stability and dominance of *Lactobacillus* [32].

Reviews focusing on the cervical microbiota and cancer further emphasize the role of endogenous hormones and menopausal status in shaping cervicovaginal communities, with menopause generally associated with reduced *Lactobacillus*, increased anaerobic taxa, and heightened inflammation, conditions that may promote neoplastic processes [22,33]. Also, a large case series of HPV-positive women with CIN and invasive cervical cancer showed that microbiome, metabolite, and cytokine interactions differed before and after menopause, with age-specific cancer-associated genera and metabolite correlations, supporting a strong effect of hormonal and menstrual status on the microenvironment of cervical neoplasia [34].

Several limitations of this study should be acknowledged. The relatively small size of the cervical cancer group may have limited statistical power for certain analyses, particularly differential abundance testing. As a consequence, the probability of detecting small effects, particularly in high-dimensional microbiome data, is reduced, and the risk of false negatives is higher.

In addition, the cross-sectional design precludes causal inference regarding whether microbiome alterations contribute to lesion progression or arise as a consequence of disease. Although this study was not designed to investigate host-related determinants, our results showed that physical activity, HPV vaccination status, and menstrual cycle phase reached nominal significance. On the other hand, their modest effect sizes indicated a limited contribution to overall microbiome variation. Several other factors showed borderline associations, which may be biologically plausible but cannot be interpreted definitively due to limited statistical power. Larger, adequately powered studies are needed to confirm these findings.

In summary, our data support a model in which progression to cervical cancer is associated with a major reorganization of the cervicovaginal microbiome, characterized by loss of *Lactobacillus* dominance, increased diversity, and consolidation of complex anaerobic networks. These changes appear to reflect a shift in community state associated with advanced disease rather than a gradual continuum across early lesion stages. Finally, the use of compositional log-ratio indices provides a quantitative measure of the *Lactobacillus*-to-anaerobe balance, and it could be further studied as a potentially more robust marker of dysbiosis than relative abundance alone.

Thus, our findings highlight the cervicovaginal microbiome as a potential biomarker of disease progression and a candidate target for adjunctive strategies in cervical cancer prevention and management. However, high inter-individual variability, temporal instability influenced by hormonal and environmental factors, and lack of standardized sampling and analytical pipelines limit immediate clinical translation of vaginal microbiota.

## 5. Conclusions

This study demonstrated that cervical disease progression is accompanied by a structured reorganization of the cervicovaginal microbiome. While early and intermediate lesion stages of cervical dysplasia (LSIL and HSIL) are characterized by heterogeneous and partially destabilized microbial communities, the transition to invasive cervical cancer is marked by a distinct shift toward highly diverse, anaerobe-rich, microbial environment.

Our analyses identified loss of *Lactobacillus* dominance as a central feature of this transition, best captured through compositional and community-level approaches rather than single-taxon comparisons.

These findings suggest that microbiome alterations may reflect and potentially reinforce the pathological microenvironment characteristic of invasive carcinoma.

## Figures and Tables

**Table 1:** Baseline characteristics of the included patients.

Variable	Category/Level	Normal (*n* = 26)	LSIL (*n* = 25)	HSIL (*n* = 25)	CCU (*n* = 10)	*p* Value
Age (years)	—	42.35 ± 9.40	35.40 ± 8.86	37.56 ± 9.88	45.10 ± 8.80	0.0109
Physical activity	None	1 (3.85)	0 (0.00)	2 (8.00)	1 (10.00)	0.783
	Rare	9 (34.62)	13 (52.00)	12 (48.00)	4 (40.00)	
	1–2 times/week	9 (34.62)	6 (24.00)	4 (16.00)	3 (30.00)	
	3–5 times/week	4 (15.38)	3 (12.00)	2 (8.00)	0 (0.00)	
	Daily	3 (11.54)	3 (12.00)	5 (20.00)	2 (20.00)	
Menstrual cycle phase	Follicular	3 (13.04)	8 (33.33)	8 (40.00)	1 (16.67)	0.345
	Luteal	13 (56.52)	8 (33.33)	5 (25.00)	3 (50.00)	
	Menstrual	4 (17.39)	6 (25.00)	2 (10.00)	1 (16.67)	
	Ovulatory	3 (13.04)	2 (8.33)	5 (25.00)	1 (16.67)	
Use of vaginal hygiene products	Yes	8 (30.77)	5 (20.00)	9 (36.00)	4 (40.00)	0.554
	No	18 (69.23)	20 (80.00)	16 (64.00)	6 (60.00)	
History of hormonal disorders	None	23 (88.46)	24 (96.00)	22 (88.00)	9 (90.00)	0.745
	Yes	3 (11.54)	1 (4.00)	3 (12.00)	1 (10.00)	
High-risk HPV infection	Negative	14 (53.85)	3 (12.00)	0 (0.00)	0 (0.00)	<0.001
	Positive	12 (46.15)	22 (88.00)	25 (100.00)	10 (100.00)	
HPV vaccination status	Yes	4 (15.38)	10 (40.00)	7 (28.00)	0 (0.00)	0.049
	No	22 (84.62)	15 (60.00)	18 (72.00)	10 (100.00)	
Number of HPV vaccine doses (categorical)	0	21 (80.77)	15 (60.00)	18 (72.00)	10 (100.00)	0.581
	1	0 (0.00)	1 (4.00)	1 (4.00)	0 (0.00)	
	2	3 (11.54)	5 (20.00)	3 (12.00)	0 (0.00)	
	3	2 (7.69)	4 (16.00)	3 (12.00)	0 (0.00)	
Smoking history	Never	16 (61.54)	15 (60.00)	10 (40.00)	4 (40.00)	0.111
	Former	3 (11.54)	5 (20.00)	12 (48.00)	4 (40.00)	
	Current	7 (26.92)	5 (20.00)	3 (12.00)	2 (20.00)	
Dietary pattern	Mixed	21 (80.77)	23 (92.00)	24 (96.00)	8 (80.00)	0.367
	High fiber	3 (11.54)	2 (8.00)	0 (0.00)	1 (10.00)	
	High sugar	2 (7.69)	0 (0.00)	1 (4.00)	1 (10.00)	
Frequency of contraceptive use	Never	5 (19.23)	10 (40.00)	12 (48.00)	8 (80.00)	0.099
	Rare	5 (19.23)	2 (8.00)	3 (12.00)	0 (0.00)	
	Often	13 (50.00)	9 (36.00)	6 (24.00)	2 (20.00)	
	Always	3 (11.54)	4 (16.00)	4 (16.00)	0 (0.00)	
Hormonal contraceptive use	Yes	8 (30.77)	3 (12.00)	7 (28.00)	4 (40.00)	0.266
	No	18 (69.23)	22 (88.00)	18 (72.00)	6 (60.00)	
History of STIs	Yes	1 (3.85)	0 (0.00)	2 (8.00)	0 (0.00)	0.426
	No	25 (96.15)	25 (100.00)	23 (92.00)	10 (100.00)	
Recent vaginal symptoms	None	16 (61.54)	12 (48.00)	11 (44.00)	5 (50.00)	0.829
	Any symptom	10 (38.46)	13 (52.00)	14 (56.00)	5 (50.00)	
Number of sexual partners	—	3.12 ± 3.14	3.48 ± 2.28	3.76 ± 2.62	2.80 ± 1.99	0.733
Number of pregnancies	—	1.00 ± 0.94	0.88 ± 0.97	0.88 ± 0.95	1.50 ± 1.65	0.411
Immunodepression	Yes	1 (3.85)	1 (4.00)	0 (0.00)	0 (0.00)	0.704
Immunosuppressive medication	Yes	1 (3.85)	1 (4.00)	0 (0.00)	0 (0.00)	0.704
Diabetes mellitus	Yes	0 (0.00)	1 (4.00)	0 (0.00)	0 (0.00)	0.481
Recurrent vaginal infections	Yes	9 (34.62)	7 (28.00)	8 (32.00)	2 (20.00)	0.842
Autoimmune/chronic diseases	Yes	1 (3.85)	2 (8.00)	0 (0.00)	0 (0.00)	0.426
Occupational exposure	Yes	1 (3.85)	1 (4.00)	0 (0.00)	0 (0.00)	0.704
Recreational drug use	Yes	1 (3.85)	0 (0.00)	1 (4.00)	0 (0.00)	0.704
Alcohol consumption	Yes	2 (7.69)	0 (0.00)	3 (12.00)	2 (20.00)	0.203
Use of probiotics/prebiotics	Yes	8 (30.77)	5 (20.00)	1 (4.00)	2 (20.00)	0.277
Perceived stress level	Low	4 (15.38)	3 (12.00)	2 (8.00)	1 (10.00)	0.991
	Moderate	11 (42.31)	12 (48.00)	12 (48.00)	5 (50.00)	
	High	9 (34.62)	8 (32.00)	7 (28.00)	3 (30.00)	
	Very high	2 (7.69)	2 (8.00)	4 (16.00)	1 (10.00)	

CCU, cervical cancer (invasive carcinoma); HSIL, high-grade squamous intraepithelial lesion; LSIL, low-grade squamous intraepithelial lesion; HPV, human papillomavirus; STIs, sexually transmitted infections.

**Table 3:** Alpha diversity—pairwise Mann–Whitney + FDR + Cliff’s delta.

Alpha Diversity Metric	Group 1	Group 2	*p* Value	FDR-Adjusted *p* Value	Cliff’s Delta	Effect Size Magnitude
Richness	Normal	LSIL	0.273	0.427	−0.19	Small
	Normal	HSIL	0.740	0.740	−0.06	Negligible
	Normal	CCU	0.0157	0.0471	−0.53	Large
	LSIL	HSIL	0.321	0.427	0.17	Small
	LSIL	CCU	0.356	0.427	−0.21	Small
	HSIL	CCU	0.0152	0.0471	−0.54	Large
Shannon index	Normal	LSIL	0.288	0.375	−0.18	Small
	Normal	HSIL	0.312	0.375	−0.17	Small
	Normal	CCU	0.0000047	0.000028	−0.90	Large
	LSIL	HSIL	0.992	0.992	−0.00	Negligible
	LSIL	CCU	0.0027	0.0068	−0.67	Large
	HSIL	CCU	0.0034	0.0068	−0.65	Large

CCU, cervical cancer (invasive carcinoma); HSIL, high-grade squamous intraepithelial lesion; LSIL, low-grade squamous intraepithelial lesion; FDR, false discovery rate.

**Table 4:** Pairwise comparisons of vaginal microbiome alpha diversity across cervical lesion severity.

Comparison	Group 1	Group 2	*p* Value	FDR-Adjusted *p* Value
Normal vs. LSIL	Normal	LSIL	0.288	0.375
Normal vs. HSIL	Normal	HSIL	0.312	0.375
Normal vs. CCU	Normal	CCU	0.000047	0.000280
LSIL vs. HSIL	LSIL	HSIL	0.992	0.992
LSIL vs. CCU	LSIL	CCU	0.0027	0.0068
HSIL vs. CCU	HSIL	CCU	0.0034	0.0068

CCU, cervical cancer (invasive carcinoma); HSIL, high-grade squamous intraepithelial lesion; LSIL, low-grade squamous intraepithelial lesion; FDR, false discovery rate.

**Table 5:** Global PERMANOVA of vaginal microbiome composition across diagnostic categories.

Metric	Value
Distance metric	Aitchison (CLR-Euclidean)
Number of samples	82
Number of groups	4
Pseudo-F	2.43
*p* value	0.0006
Permutations	9999

CLR, centered log-ratio.

**Table 6:** Pairwise PERMANOVA comparisons of vaginal microbiome composition.

Group 1	Group 2	Pseudo-F	R^2^	*p* Value	FDR-Adjusted *p* Value
Normal	LSIL	1.61	0.034	0.0836	0.1003
Normal	HSIL	2.67	0.054	0.0046	0.0138
Normal	CCU	5.50	0.143	0.0001	0.0006
LSIL	HSIL	1.25	0.027	0.2228	0.2228
LSIL	CCU	2.10	0.063	0.0251	0.0377
HSIL	CCU	2.38	0.069	0.0148	0.0296

CCU, cervical cancer (invasive carcinoma); HSIL, high-grade squamous intraepithelial lesion; LSIL, low-grade squamous intraepithelial lesion; FDR, false discovery rate.

**Table 7:** Mean Aitchison distances between vaginal microbiome profiles across diagnostic categories.

Group	Normal	LSIL	HSIL	CCU
Normal	8.47	10.74	9.91	14.98
LSIL	10.74	12.77	11.83	15.98
HSIL	9.91	11.83	10.78	14.88
CCU	14.98	15.98	14.88	17.33

CCU, cervical cancer (invasive carcinoma); HSIL, high-grade squamous intraepithelial lesion; LSIL, low-grade squamous intraepithelial lesion .

**Table 8:** Multivariate dispersion (PERMDISP) across diagnostic categories.

Diagnosis	Mean Dispersion
Normal	5.86
LSIL	8.69
HSIL	7.35
CCU	11.42

CCU, cervical cancer (invasive carcinoma); HSIL, high-grade squamous intraepithelial lesion; LSIL, low-grade squamous intraepithelial lesion.

**Table 9:** PERMANOVA of host factors and vaginal microbiome composition.

Factor	Pseudo-F	*p* Value
Physical Activity	1.835674	0.007
HPV Vaccination	2.594235	0.014
Current Menstrual Cycle Phase	1.669606	0.046
Use of Vaginal Hygiene Products	1.988036	0.060
High-Risk HPV Status	1.841416	0.081
History of Hormonal Disorders	1.623280	0.084
Number of HPV Vaccine Doses	1.359499	0.139
Smoking History	1.291031	0.189
Diet	1.134758	0.324
Use of Hormonal Contraceptives	1.032019	0.359
Regular Menstrual Cycle	0.927137	0.450
Frequency of Contraceptive Use	0.995913	0.453
History of STIs	0.876504	0.490
Recent Vaginal Infection Symptoms	0.995409	0.497
Age	0.997232	0.506
Number of Sexual Partners	0.965878	0.569
Immunodeficiency	0.762004	0.573
Immunosuppressive or Immune-Modulating Medication	0.727251	0.603
Number of Pregnancies	0.894492	0.607
Diabetes	0.565838	0.620
History of Recurrent Vaginal Infections	0.690814	0.689
Occupational Exposure to Agents	0.657205	0.711
Recreational Drug Use	0.621749	0.729
Use of Probiotics or Prebiotics	0.642788	0.760
Autoimmune or Chronic Diseases	0.576460	0.800
Alcohol Consumption	0.527939	0.851
Stress Level	0.688064	0.875

HPV, human papillomavirus; STIs, sexually transmitted infections.

**Table 10:** Relative abundance and prevalence of dominant vaginal bacterial genera across cervical lesion severity.

Genus	Normal Mean %	Normal Median %	Normal Prev %	LSIL Mean %	LSIL Median %	LSIL Prev %	HSIL Mean %	HSIL Median %	HSIL Prev %	CCU Mean %	CCU Median %	CCU Prev %
*Lactobacillus*	90.58	98.77	100.00	83.48	96.33	95.65	66.88	96.08	91.67	19.52	5.02	80.00
*Streptococcus*	4.83	0.00	44.00	2.44	0.00	47.83	5.91	0.00	45.83	17.44	0.07	60.00
*Limosilactobacillus*	2.65	0.26	60.00	2.87	0.00	47.83	0.67	0.00	33.33	0.68	0.00	30.00
*Dialister*	0.52	0.00	48.00	2.24	0.03	52.17	1.72	0.00	41.67	0.69	0.00	40.00
*Prevotella*	0.28	0.00	28.00	1.82	0.00	47.83	0.56	0.00	25.00	4.06	1.39	70.00
*Staphylococcus*	0.27	0.00	28.00	1.17	0.00	39.13	0.08	0.00	33.33	1.32	0.00	30.00
*Hoylesella*	0.13	0.00	20.00	0.57	0.00	26.09	0.28	0.00	8.33	0.20	0.00	40.00
*Anaerococcus*	0.10	0.00	28.00	0.29	0.01	60.87	1.81	0.00	33.33	5.16	0.88	70.00
*Peptoniphilus*	0.10	0.00	40.00	0.52	0.00	43.48	1.66	0.00	33.33	1.83	0.66	70.00
*Fenollaria*	0.08	0.00	16.00	0.60	0.00	17.39	0.38	0.00	12.50	0.07	0.00	20.00
*Campylobacter*	0.08	0.00	24.00	0.24	0.00	39.13	0.73	0.00	16.67	0.36	0.00	50.00
*Finegoldia*	0.07	0.00	48.00	0.25	0.01	56.52	3.33	0.00	41.67	1.71	0.07	60.00
*Fusobacterium*	0.02	0.00	20.00	0.58	0.00	17.39	1.95	0.00	16.67	4.19	0.00	30.00
*Enterococcus*	0.00	0.00	4.00	0.13	0.00	21.74	0.03	0.00	4.17	6.13	0.00	30.00
*Peptostreptococcus*	0.00	0.00	8.00	0.59	0.00	17.39	1.29	0.00	20.83	7.93	0.02	50.00
*Fannyhessea*	0.00	0.00	0.00	0.24	0.00	21.74	3.93	0.00	25.00	5.07	0.00	10.00
*Pasteurella*	0.00	0.00	0.00	0.00	0.00	0.00	0.56	0.00	4.17	6.88	0.00	10.00

CCU, cervical cancer (invasive carcinoma); HSIL, high-grade squamous intraepithelial lesion; LSIL, low-grade squamous intraepithelial lesion.

**Table 11:** Global and pairwise ANCOM-BC2 results for taxa of interest.

Genus	Global *p* Value	Global FDR (q)	CCU vs. Normal logFC	CCU vs. Normal q	CCU vs. LSIL logFC	CCU vs. LSIL q	HSIL vs. LSIL logFC	HSIL vs. LSIL q	LSIL vs. Normal logFC	LSIL vs. Normal q
*Lactobacillus*	0.0038	0.369	−5.47	0.0596	−4.49	0.311	−1.38	0.647	−0.98	0.558
*Prevotella*	0.0166	0.369	2.76	0.160	1.47	0.929	−1.22	0.422	1.30	0.558
*Dialister*	0.306	0.450	−0.24	0.409	−0.98	0.929	−1.17	0.422	0.74	0.657
*Staphylococcus*	0.503	0.604	−0.26	0.391	−0.85	0.929	−0.82	0.479	0.58	0.657

CCU, cervical cancer (invasive carcinoma); HSIL, high-grade squamous intraepithelial lesion; LSIL, low-grade squamous intraepithelial lesion; FDR, false discovery rate.

**Table 12:** Distribution of *Lactobacillus*-to-anaerobe log-ratio across cervical disease categories.

Diagnosis	Median	Q1–Q3
Normal	5.06	4.41–6.36
LSIL	3.57	3.05–5.22
HSIL	4.34	0.21–5.07
CCU	0.51	−0.63–1.26

CCU, cervical cancer (invasive carcinoma); HSIL, high-grade squamous intraepithelial lesion; LSIL, low-grade squamous intraepithelial lesion.

**Table 13:** Pairwise comparisons of log-ratio values across diagnostic categories.

Group 1	Group 2	*p* Value	FDR-Adjusted *p* Value	Cliff’s Delta	Effect Size
Normal	LSIL	0.0149	0.0223	0.41	Medium
Normal	HSIL	0.0135	0.0223	0.41	Medium
Normal	CCU	8.7 × 10^−5^	5.2 × 10^−4^	0.86	Large
LSIL	HSIL	0.710	0.710	0.07	Negligible
LSIL	CCU	0.0040	0.0120	0.64	Large
HSIL	CCU	0.0726	0.0871	0.40	Medium

CCU, cervical cancer (invasive carcinoma); HSIL, high-grade squamous intraepithelial lesion; LSIL, low-grade squamous intraepithelial lesion; FDR, false discovery rate.

**Table 14:** Non-parametric pairwise comparisons of log-ratio values.

Group 1	Group 2	*p* Value	FDR-Adjusted *p* Value
Normal	LSIL	0.117	0.701
Normal	HSIL	0.171	1.000
Normal	CCU	0.00010	0.00060
LSIL	HSIL	0.856	1.000
LSIL	CCU	0.00107	0.00643
HSIL	CCU	0.0120	0.0717

CCU, cervical cancer (invasive carcinoma); HSIL, high-grade squamous intraepithelial lesion; LSIL, low-grade squamous intraepithelial lesion; FDR, false discovery rate.

**Table 15:** Strongest genus–genus correlations within each diagnostic category.

Normal			
Genus 1	Genus 2	Spearman’s ρ	*p* Value
*Peptostreptococcus*	*Escherichia*	0.92	<0.001
*Veillonella*	*Shigella*	0.80	<0.001
*Prevotella*	*Hoylesella*	0.77	<0.001
*Anaerococcus*	*Hoylesella*	0.76	<0.001
*Anaerococcus*	*Peptoniphilus*	0.74	<0.001
LSIL			
*Shigella*	*Escherichia*	0.78	<0.001
*Dialister*	*Anaerococcus*	0.71	<0.001
*Dialister*	*Prevotella*	0.69	<0.001
*Peptostreptococcus*	*Fusobacterium*	0.60	<0.01
*Prevotella*	*Anaerococcus*	0.60	<0.01
HSIL			
*Shigella*	*Escherichia*	0.87	<0.001
*Fusobacterium*	*Campylobacter*	0.80	<0.001
*Peptoniphilus*	*Finegoldia*	0.74	<0.001
*Anaerococcus*	*Campylobacter*	0.74	<0.001
*Peptostreptococcus*	*Fusobacterium*	0.71	<0.001
Invasive carcinoma			
*Shigella*	*Escherichia*	1.00	<0.001
*Dialister*	*Hoylesella*	0.99	<0.001
*Veillonella*	*Pseudomonas*	0.94	<0.001
*Anaerococcus*	*Peptoniphilus*	0.93	<0.001
*Ureaplasma*	*Staphylococcus*	0.87	<0.01

**Table 16:** Correlation network analysis.

Group	Nodes and Edges	Density	Genus 1	Genus 2	Spearman ρ	*p* Value	q Value
Normal	Genera retained after filtering (prevalence ≥ 0.3, max 20): 7 Filtered edges (|ρ| ≥ 0.6, q < 0.05): 1	0.047	*Dialister*	*Peptoniphilus*	0.68615385	<0.001	0.003
LSIL	Genera retained after filtering (prevalence ≥ 0.3, max 20): 15 Filtered edges (|ρ| ≥ 0.6, q < 0.05): 3	0.028	*Escherichia*	*Shigella*	0.77569170	<0.001	0.001
			*Dialister*	*Anaerococcus*	0.70750988	<0.001	0.008
			*Dialister*	*Prevotella*	0.68873518	<0.001	0.009
HSIL	Genera retained after filtering (prevalence ≥ 0.3, max 20): 10 Filtered edges (|ρ| ≥ 0.6, q < 0.05): 2	0.044	*Finegoldia*	*Peptoniphilus*	0.74000000	<0.001	0.001
			*Dialister*	*Peptoniphilus*	0.68695652	<0.001	0.004
CCU	Genera retained after filtering (prevalence ≥ 0.4, max 20): 11 Filtered edges (|ρ| ≥ 0.7, q < 0.05): 4	0.072	*Dialister*	*Hoylesella*	0.98787879	<0.001	<0.001
			*Anaerococcus*	*Peptoniphilus*	0.92727273	<0.001	0.003
			*Anaerococcus*	*Pseudomonas*	−0.85454545	<0.001	0.022
			*Peptoniphilus*	*Campylobacter*	0.85454545	<0.001	0.0225

CCU, cervical cancer (invasive carcinoma); HSIL, high-grade squamous intraepithelial lesion; LSIL, low-grade squamous intraepithelial lesion.

## Data Availability

The data presented in this study are available on request from the corresponding author. The data are not publicly available due to local regulations.

## Abbreviations

The following abbreviations are used in this manuscript:

ANCOM-BC2	Analysis of Composition of Microbiomes with Bias Correction 2
BMI	Body mass index
CCU	Cervical cancer (invasive carcinoma)
CI	Confidence interval
CIN	Cervical intraepithelial neoplasia
CLR	Centered log-ratio
CST	Community state type
DNA	Deoxyribonucleic acid
FDR	False discovery rate
HPV	Human papillomavirus
HR-HPV	High-risk human papillomavirus
HSIL	High-grade squamous intraepithelial lesion
logFC	Log fold change
LSIL	Low-grade squamous intraepithelial lesion
NILM	Negative for intraepithelial neoplasia
ONT	Oxford Nanopore Technologies
OR	Odds ratio
PCoA	Principal Coordinates Analysis
PERMANOVA	Permutational multivariate analysis of variance
PERMDISP	Permutational analysis of multivariate dispersions
Q1–Q3	First to third quartile (interquartile range)
q	FDR-adjusted p value
R^2^	Coefficient of determination
ROC	Receiver operating characteristic
SD	Standard deviation
STIs	Sexually transmitted infections
ρ (rho)	Spearman’s rank correlation coefficient

## References

[1] Li Y., Song W., Gao P., Guan X., Wang B., Zhang L., Yao Y., Guo Y., Wang Y., Jiang S. (2025). Global, regional, and national burden of breast, cervical, uterine, and ovarian cancer and their risk factors among women from 1990 to 2021, and projections to 2050: Findings from the global burden of disease study 2021. BMC Cancer.

[2] Li Z., Liu P., Yin A., Zhang B., Xu J., Chen Z., Zhang Z., Zhang Y., Wang S., Tang L. (2025). Global landscape of cervical cancer incidence and mortality in 2022 and predictions to 2030: The urgent need to address inequalities in cervical cancer. Int. J. Cancer.

[3] Bowden S., Doulgeraki T., Bouras E., Markozannes G., Athanasiou A., Grout-Smith H., Kechagias K., Ellis L., Zuber V., Chadeau-Hyam M. (2023). Risk factors for human papillomavirus infection, cervical intraepithelial neoplasia and cervical cancer: An umbrella review and follow-up Mendelian randomisation studies. BMC Med..

[4] Kusakabe M., Taguchi A., Sone K., Mori M., Osuga Y. (2023). Carcinogenesis and management of human papillomavirus-associated cervical cancer. Int. J. Clin. Oncol..

[5] Bautista J., Altamirano-Colina A., López-Cortés A. (2025). The vaginal microbiome in HPV persistence and cervical cancer progression. Front. Cell. Infect. Microbiol..

[6] France M., Alizadeh M., Brown S., Ma B., Ravel J. (2022). Towards a deeper understanding of the vaginal microbiota. Nat. Microbiol..

[7] Chee W.J.Y., Chew S., Than L. (2020). Vaginal microbiota and the potential of Lactobacillus derivatives in maintaining vaginal health. Microb. Cell Fact..

[8] Delgado-Diaz D., Jesaveluk B., Hayward J., Tyssen D., Alisoltani A., Potgieter M., Bell L., Ross E., Iranzadeh A., Allali I. (2022). Lactic acid from vaginal microbiota enhances cervicovaginal epithelial barrier integrity by promoting tight junction protein expression. Microbiome.

[9] Zhang Z., Zheng K., Zhang Z., Cao L., Lin L., Sun W., Qiu F. (2025). Lactobacillus gasseri LGV03-derived indole-3-lactic acid ameliorates immune response by activating aryl hydrocarbon receptor. Microb. Cell Fact..

[10] Mahajan G., Doherty E., To T., Sutherland A., Grant J., Junaid A., Gulati A., LoGrande N., Izadifar Z., Timilsina S. (2022). Vaginal microbiome-host interactions modeled in a human vagina-on-a-chip. Microbiome.

[11] Alizhan D., Ukybassova T., Bapayeva G., Aimagambetova G., Kongrtay K., Kamzayeva N., Terzic M. (2025). Cervicovaginal Microbiome: Physiology, Age-Related Changes, and Protective Role Against Human Papillomavirus Infection. J. Clin. Med..

[12] Pai H., Baid R., Palshetkar N., Pai R., Pai A., Palshetkar R. (2025). Role of Vaginal and Gut Microbiota in Human Papillomavirus (HPV) Progression and Cervical Cancer: A Systematic Review of Microbial Diversity and Probiotic Interventions. Cureus.

[13] Gao Y.-Y., Li X.-X., Zuo H., Zhang P. (2025). Analysis of the Relationship Between Cervical Cancer Progression and the Microbiome. Int. J. Gen. Med..

[14] Teka B., Yoshida-Court K., Firdawoke E., Chanyalew Z., Gizaw M., Addissie A., Mihret A., Colbert L., Napravnik T., Alam M.E. (2023). Cervicovaginal Microbiota Profiles in Precancerous Lesions and Cervical Cancer among Ethiopian Women. Microorganisms.

[15] Zhai Q., Zhao L., Wang M., Li L., Li L., Ye M., Li M., Xu C., Meng Y. (2025). Integrated analysis of microbiome and metabolome reveals insights into cervical neoplasia aggravation in a Chinese cohort. Front. Cell. Infect. Microbiol..

[16] Sims T., Biegert G., Ramogola-Masire D., Ngoni K., Solley T., Ning M., Alam M.E., Mezzari M., Petrosino J., Zetola N. (2020). Tumor microbial diversity and compositional differences among women in Botswana with high-grade cervical dysplasia and cervical cancer. Int. J. Gynecol. Cancer.

[17] Qin L., Sun T., Li X., Zhao S., Liu Z., Zhang C., Jin C., Xu Y., Gao X., Cao Y. (2025). Population-level analyses identify host and environmental variables influencing the vaginal microbiome. Signal Transduct. Target. Ther..

[18] Li X., Xiang F., Liu T., Chen Z., Zhang M., Li J., Kang X., Wu R. (2024). Leveraging existing 16S rRNA gene surveys to decipher microbial signatures and dysbiosis in cervical carcinogenesis. Sci. Rep..

[19] Liu Y., Wang S., Liu J., Su M., Diao X., Liang X., Zhang J., Wang Q., Zhan Y. (2023). Characteristics of vaginal microbiota in various cervical intraepithelial neoplasia: A cross-sectional study. J. Transl. Med..

[20] So K.A., Yang E.J., Kim N.R., Hong S.R., Lee J.H., Hwang C.S., Shim S.H., Lee S.J., Kim T.J. (2020). Changes of vaginal microbiota during cervical carcinogenesis in women with human papillomavirus infection. PLoS ONE.

[21] Wu S., Ding X., Kong Y., Acharya S., Wu H., Huang C., Liang Y., Nong X., Chen H. (2021). The feature of cervical microbiota associated with the progression of cervical cancer among reproductive females. Gynecol. Oncol..

[22] Wen Q., Wang S., Min Y., Liu X., Fang J., Lang J., Chen M. (2025). Associations of the gut, cervical, and vaginal microbiota with cervical cancer: A systematic review and meta-analysis. BMC Women’s Health.

[23] Pu X., Wang X., Wang J., Gu Z., Zhu H., Li C. (2025). Microbial and metabolic profiles associated with HPV infection and cervical intraepithelial neoplasia: A multi-omics study. Microbiol. Spectr..

[24] Rashwan H.H., Ali M.H., Mostafa M.M., Ramadan R., Mysara M. (2025). Insights into the tripartite relationship between cervical cancer, human papillomavirus, and the vaginal microbiome: A mega-analysis. Hum. Genom..

[25] Passarelli G.V., Whang S.N., Gilbert N.M., Hu J. (2026). The vaginal microbiome, papillomavirus infection, and cervical cancer: Established associations in search of model systems and mechanistic answers. mBio.

[26] Schellekens H.C.J., Schmidt L.M.S., Morré S.A., van Esch E.M.G., de Vos van Steenwijk P.J. (2025). Vaginal Microbiota and Local Immunity in HPV-Induced High-Grade Cervical Dysplasia: A Narrative Review. Int. J. Mol. Sci..

[27] Lagunas-Cruz M.D.C., Valle-Mendiola A., Soto-Cruz I. (2026). The Vaginal Microbiome and Host Health: Implications for Cervical Cancer Progression. Int. J. Mol. Sci..

[28] Jimenez N.R., Mancilla V., Łaniewski P., Herbst-Kralovetz M.M. (2025). Immunometabolic Contributions of Atopobiaceae Family Members in Human Papillomavirus Infection, Cervical Dysplasia, and Cancer. J. Infect. Dis..

[29] Chen Y.-L., Qiu X., Wang W., Li D., Wu A., Hong Z., Di W., Qiu L. (2020). Human papillomavirus infection and cervical intraepithelial neoplasia progression are associated with increased vaginal microbiome diversity in a Chinese cohort. BMC Infect. Dis..

[30] Mulato-Briones I.B., Rodriguez-Ildefonso I.O., Jiménez-Tenorio J.A., Cauich-Sánchez P.I., Méndez-Tovar M.D.S., Aparicio-Ozores G., Bautista-Hernández M.Y., González-Parra J.F., Cruz-Hernández J., López-Romero R. (2024). Cultivable Microbiome Approach Applied to Cervical Cancer Exploration. Cancers.

[31] Mitra A., MacIntyre D., Ntritsos G., Smith A., Tsilidis K., Marchesi J., Bennett P., Moscicki A., Kyrgiou M. (2020). The vaginal microbiota associates with the regression of untreated cervical intraepithelial neoplasia 2 lesions. Nat. Commun..

[32] Kyrgiou M., Mitra A., Moscicki A. (2017). Does the vaginal microbiota play a role in the development of cervical cancer?. Transl. Res..

[33] Li X., Wu J., Wu Y., Duan Z., Luo M., Li L., Li S., Jia Y. (2023). Imbalance of Vaginal Microbiota and Immunity: Two Main Accomplices of Cervical Cancer in Chinese Women. Int. J. Women’s Health.

[34] Kawasaki R., Kukimoto I., Nishio E., Otani S., Nishizawa H., Maeda Y., Iwata A., Fujii T. (2025). Distinct cervical microbiome and metabolite profiles before and after menopause: Implications for cervical cancer progression. Front. Cell. Infect. Microbiol..

